# Pontocerebellar hypoplasia type 2D in two Acadian families: functional RNA characterization of the recurrent *SEPSECS* c.388+3A>G variant

**DOI:** 10.3389/fgene.2026.1954385

**Published:** 2026-10-30

**Authors:** Nadia Bouhamdani, Raphael Fournier, Sarah Dyack, Kayla Roddick, Elina Nikkola, ZhenBin Chen, Neta Pipko, Yijing Liang, Ashish R. Deshwar, Gregory Costain, Inas Al-Younis, Mouna Ben Amor

**Affiliations:** 1 Vitalité Health Network, Moncton, NB, Canada; 2 University of Sherbrooke, Sherbrooke, QC, Canada; 3 University of Moncton, Department of Chemistry and Biochemistry, Moncton, NB, Canada; 4 Atlantic Cancer Research Institute, Moncton, NB, Canada; 5 Centre de Formation Médicale du Nouveau-Brunswick, Moncton, NB, Canada; 6 IWK Health Centre, Division of Medical Genetics, Halifax, NS, Canada; 7 Dalhousie University, Department of Pediatrics, Halifax, NS, Canada; 8 Stan Cassidy Centre for Rehabilitation, Fredericton, NB, Canada; 9 Fulgent Genetics, El Monte, CA, United States; 10 Program in Genetics and Genome Biology, SickKids Research Institute, Toronto, ON, Canada; 11 The Centre for Computational Medicine, SickKids Research Institute, Toronto, ON, Canada; 12 Division of Clinical & Metabolic Genetics, Hospital for Sick Children, Toronto, ON, Canada; 13 Department of Molecular Genetics, University of Toronto, Toronto, ON, Canada; 14 Program in Developmental and Stem Cell Biology, SickKids Research Institute, Toronto, ON, Canada; 15 Department of Pediatrics, University of Toronto, Toronto, ON, Canada

**Keywords:** founder allele, RNA-seq, selenoprotein, SEPSECS, splice-altering variants, whole-exome, intronic variant, acadian

## Abstract

**Background:**

Pontocerebellar hypoplasia type 2D (PCH2D, OMIM #613811) is an ultra-rare autosomal recessive neurodevelopmental disorder caused by biallelic pathogenic variants in *SEPSECS*, which encodes a key enzyme required for selenoprotein biosynthesis. An extensive literature review conducted for this report identified approximately 34 genetically confirmed patients from 26 families with *SEPSECS*-related disease across the published literature, underscoring the extreme rarity of PCH2D. Intronic splice-altering variants with functional RNA confirmation remain poorly characterized, and no population-specific founder variant has been established for *SEPSECS*-related PCH2D.

**Methods:**

We performed detailed clinical, molecular, and transcriptomic analyses in three affected individuals from two unrelated Acadian families originating from the Canadian Maritime provinces. All individuals underwent exome sequencing, followed by RNA-based analyses of patient-derived samples to assess the functional impact of the recurrent intronic *SEPSECS* variant.

**Results:**

Exome sequencing identified the same homozygous intronic *SEPSECS* variant, c.388 + 3A>G, in all three affected individuals. The variant was initially classified as a variant of uncertain significance in patients 1 and 2 and as likely pathogenic in patient 3 by different diagnostic laboratories. RNA analysis demonstrated aberrant splicing with exon 3 skipping, generating an out-of-frame transcript consistent with a loss-of-function mechanism. Residual normally spliced transcript was also detected in blood-derived RNA, consistent with a partial or “leaky” splice defect at the transcript level. These findings supported pathogenic reclassification and established a molecular diagnosis of PCH2D. Clinically, the patients presented with early-onset lower-extremity spasticity, gait abnormalities, nystagmus, and mild neurodevelopmental delay, but had an attenuated clinical course compared to classical severe PCH2D.

**Conclusion:**

This case series expands the molecular and clinical spectrum of *SEPSECS*-related PCH2D by providing functional RNA evidence for a recurrent non-canonical intronic splice variant and documenting an attenuated infantile-onset presentation. The recurrence of c.388 + 3A>G in individuals of Acadian ancestry raises the possibility of a candidate Acadian founder allele. These findings underscore the importance of transcript-level analyses for interpreting intronic variants and improving diagnosis in ultra-rare neurodevelopmental disorders.

## 1 Introduction

Pontocerebellar hypoplasia (PCH) comprises a heterogeneous group of rare autosomal recessive neurodevelopmental disorders characterized by prenatal or early postnatal onset of cerebellar and pontine hypoplasia, frequently accompanied by progressive cerebral atrophy, severe developmental impairment, epilepsy, movement disorders, and early mortality in the most severe forms (Barth and hypoplasias, 1993; Barth et al., 2007; Namavar et al., 2011a; Namavar et al., 2011b; Zhao et al., 2023). With advances in molecular genetics, PCH has been subdivided into an expanding number of entities (currently more than 15 subtypes), many of which are caused by defects in fundamental cellular processes such as RNA processing, tRNA splicing, and protein translation (5–7) (Zhao et al., 2023; van Dijk et al., 2018; Budde et al., 2008). Among these disorders, pontocerebellar hypoplasia type 2 (PCH2) represents one of the best-characterized clinical subgroups and is itself genetically heterogeneous. Distinct molecular forms include PCH2A (*TSEN54,* OMIM #277470), PCH2B (*TSEN2*, OMIM #612389), PCH2C (*TSEN34,* OMIM #612390), and PCH2D (OMIM #613811), the latter resulting from biallelic pathogenic variants in the *SEPSECS* gene (Agamy et al., 2010; Anttonen et al., 2015).

The *SEPSECS* gene, located on chromosome 4p15.2, comprises 11 exons and encodes O-phosphoseryl-tRNA:selenocysteine tRNA synthase (SepSecS), a 501–amino acid enzyme that catalyzes the final and obligate step of selenocysteine biosynthesis in humans (Zhao et al., 2023; Yuan et al., 2006; Palioura et al., 2009; Iwama et al., 2016; Nicita et al., 2022). Selenocysteine, often referred to as the 21st amino acid, is co-translationally incorporated into a restricted but biologically essential group of proteins known as selenoproteins. In humans, the selenoproteome comprises 25 proteins involved in antioxidant defense, redox signaling, mitochondrial metabolism, thyroid hormone regulation, and neuronal survival (Bellinger et al., 2009; Puppala et al., 2016; Ramadesikan et al., 2022). Selenocysteine synthesis relies on a highly specialized translational pathway involving a unique tRNA (tRNA [Ser]Sec) and several dedicated enzymes. Within this pathway, SepSecS converts phosphoseryl-tRNA [Ser]Sec into selenocysteinyl-tRNA [Ser]Sec, thereby enabling selenoprotein translation (Yuan et al., 2006; Palioura et al., 2009).

*SEPSECS*-related PCH2D is exceptionally rare. Since pathogenic *SEPSECS* variants were first identified as a cause of progressive cerebellocerebral atrophy approximately 2 decades ago (Agamy et al., 2010), only a limited number of affected individuals have been reported worldwide. An extensive literature review conducted for this report identified approximately 34 genetically confirmed patients from 26 families with *SEPSECS*-related disease across the published literature, excluding the three patients from two families reported here. Table 1 summarizes 37 genetically confirmed individuals from 28 families which includes the present cases. (Table 1) (Zhao et al., 2023; van Dijk et al., 2018; Agamy et al., 2010; Anttonen et al., 2015; Iwama et al., 2016; Nicita et al., 2022; Ramadesikan et al., 2022; Ghasemi et al., 2024; Ben-Zeev et al., 2003; Makrythanasis et al., 2014; Alazami et al., 2015; Zhu et al., 2015; Pavlidou et al., 2016; Olson et al., 2017; Hengel et al., 2020; Arrudi-Moreno et al., 2021; Martínez-Martín et al., 2022; Rong et al., 2021; Schlüter et al., 2022; Pettinato et al., 2025). Most affected individuals present in the neonatal period with severe neurodevelopmental impairment, including progressive microcephaly, profound motor delay, spastic or dystonic quadriplegia, seizures, visual impairment, and feeding difficulties, defining an early-onset presentation (EOP) (Zhao et al., 2023). In contrast, a smaller subset of patients exhibits a late-onset presentation (LOP), characterized by mild developmental delay in childhood with a more indolent course and notably absent pontine atrophy on initial neuroimaging (Zhao et al., 2023; Iwama et al., 2016). In fact, in EOP, neuroimaging typically demonstrates early onset progressive cerebellar atrophy, often accompanied by cerebral atrophy, while pontine involvement may be relatively less pronounced compared with other PCH subtypes (Zhao et al., 2023). Despite the small number of reported cases, *SEPSECS*-related PCH2D demonstrates substantial phenotypic heterogeneity. This clinical variability does not appear to correlate with mutation type or position, as pathogenic *SEPSECS* variants of diverse classes are distributed throughout the gene, precluding the establishment of clear genotype–phenotype correlations (Nicita et al., 2022; Pettinato et al., 2025) (Figure 1). Consequently, the factors underlying the observed phenotypic variability remain poorly understood.

**TABLE 1:** Summary of clinical and molecular findings in previously reported and newly identified cases of SEPSECS-related PCH2D.

Families	Number of patients	Ethnicity	Sex	Variant	Protein change	Zygosity	EOP vs. LOP	MRI findings	Functional studies	Documented phenotype	ClinVar variation ID	Refs.
2	4	Iraqi/Moroccan	M	c.1001A>G	p.Y334C	H	EOP	Cerebellar/cerebral atrophy Normal at 5 m; progressive cerebellar/frontal parasylvian atrophy Normal at 5 m; progressive cerebellar/frontal parasylvian atrophy w.m.changes thin corpus callosum at 8 m; progressive cerebellar atrophy	Protein enzymatic assay confirmed loss of SepSecS catalytic activity for Y334C and A239T (as shown in ref 8)	-DD/ID (4/4) -Epilepsy (2/4) -Horizontal nystagmus (1/4) -Non-verbal (4/4) -Spastic quadriplegia (4/4) -Secondary microcephaly (4/4) -Unable to walk or sit independently (4/4)	18400, 18401	(Ben-Zeev et al., 2003), (Nejabat et al., 2021)
			F	c.1001A>G	p.Y334C	H	EOP					
			M	c.1001A>G c.715G > A	p.Y334C p.A239T	Comp. Het	EOP					
			M	c.1001A>G c.715G > A	p.Y334C p.A239T	Comp. Het	EOP					
1	3	Jordan	F	c.1466A>T	p.D489V	H	EOP	NA	No	-DD/ID (3/3) -Epilepsy (3/3) -Non-verbal chorea (3/3) -Secondary microcephaly (3/3) -Spasticity (3/3)	190145	(Makrythanasis et al., 2014)
			F	c.1466A>T	p.D489V	H	EOP	NA				
			F	c.1466A>T	p.D489V	H	EOP	NA				
1	1	Arabian/Saudi	Unknown	*c.1028_1120+1del* Reported as c.1027_1120del *In* (Alazami et al., 2015)	p.Q343Lfs*2	H	EOP	NA	No	-Dysarthria -Nystagmus -Spasticity	183337	(Alazami et al., 2015)
3	4	Finnish	M	c.974C>G c.1287C>A	p.T325S p.T429*	Comp. Het	EOP	Early w.m.changes; progressive cerebellar/cerebral atrophy Early w.m.changes; progressive cerebellar/cerebral atrophy Progressive cerebellar and cerebral atrophy NA	Protein enzymatic assay confirmed: reduced enzymatic activity (T325S), absent truncated protein (Y429*) and decreased brain selenoproteins	-Central blindness (4/4) -DD/ID (4/4) -Epilepsy (1/4) -Infantile spasms (2/4) -Irritability (3/4) -Opisthotonus (3/4) -Spasticity (4/4)	4884327, 3780591	(Anttonen et al., 2015)
			F	c.974C>G c.1287C>A	p.T325S p.T429*	Comp. Het	EOP					
			F	c.974C>G 1287C>A	p.T325S p.T429*	Comp. Het NA	EOP					
			F	Sister of patient 3 not tested	NA		EOP					
1	1	Unknown	F	c.1A>G c.388 + 3A>G	p.M1? -	Comp. Het	EOP	NA	No	-DD -Hypotonia -Microcephaly	1223757, 378569	(Zhu et al., 2015)
1	1	Unknown	Unknown	c.388 + 3A>G c.805-1G>T *c.805-1G>T was *reported in* (Moreno-De-Luca et al., 2021) *as c.805-1- > G; likely formatting error*	- -	Comp. Het	Unknown	NA	No	- Cerebral palsy	378569, 3590471	(Moreno-De-Luca et al., 2021)
2	2	Japanese	F	c.356A>G c.77delG	p.N119S p.R26Pfs*42	Comp. Het	LOP	Normal at 2 y; progressive cerebellar/frontoparietal atrophy	No	-Ataxic gait (2/2) -DD (1/2) -Nystagmus (1/2) -Secondary microcephaly (2/2) -Spasticity (2/2) -Slurred speech (2/2)	4884329, 4884330, 1329502	(Iwama et al., 2016)
			F	c.356A>G c.467G>A	p.N119S p.R156Q	Comp. Het	LOP	Normal at 5 y; progressive cerebellar/cerebral atrophy	No			
1	1	Arabian	M	c.1000T>C *Reported as c.1001T>C in (Pavlidou et al., 2016)	p.Y334H	H	EOP	Ventricular widening at 18 m; progressive cerebellar/cerebral atrophy and optic nerve atrophy	No	-Secondary microcephaly -Spastic quadriplegia -Optic nerve atrophy -Hypotonia	800530	(Pavlidou et al., 2016)
1	1	American	M	c.176C>T	p.A59V	H	EOP	Cerebral more than cerebellar volume loss, delayed dysmyelination, pontine hypoplasia	No	-DD -Epileptic encephalopathy -Hypotonia - Microcephaly	4884331	(Olson et al., 2017)
1	1	Dutch	F	c.1321G>A	p.G441R	H	LOP	Progressive cerebellar atrophy	No	-Cerebellar ataxia -Cognitive impairment -Horizontal nystagmus -Microcephaly	3256764	(van Dijk et al., 2018)
1	2	Arabian	Unknown	c.181A>G	p.M61V	H	EOP	NA	No	-DD/ID (2/2)	810638	(Hengel et al., 2020)
			Unknown	c.181A>G	p.M61V	H	EOP	NA				
1	1	Moroccan	F	c.114 + 3A>G	-	H	EOP	Normal at 14 m; progressive ponto-cerebellar atrophy	No	-Able to say few words -Convergent nystagmus -DD -Secondary microcephaly -Spasticity -Tetraplegia	984624	(Arrudi-Moreno et al., 2021)
1	1	Italian	M	c.865C>T c.1297T>C	p.P289S p.Y433H	Comp. Het	LOP	Mild frontotemporal cerebral atrophy; thin corpus callosum no cerebellar/brainstem anomalies	No	-Bradyphrenia -Bradykinesia -Dysarthria -Dystonia -Microcephaly -Nystagmus -Optic nerve atrophy -Spastic paraparesis	4884332, 4884333	(Nicita et al., 2022)
1	2	Spanish	M	c.1321G>A	p.G441R	H	LOP	Mild atrophy of cerebellar vermis	No	-Cerebellar ataxia (2/2) -Convergent strabismus (1/2) -Fine motor disability (1/2) -ID (2/2) -Language impairment (2/2) -Nystagmus (1/2) -Pyramidal signs (1/2) -Scanning speech (2/2)	3256764	(Martínez-Martín et al., 2022)
			M	c.1321G>A	p.G441R	H	LOP	Normal				
1	2	Unknown	F	c.1A>T c.846G>A	p.M1? p.L282	Comp. Het	EOP	w.m.changes at birth; progressive cerebral/ponto-cerebellar atrophy	RT-PCR and RNA seq. confirms that variant c.846G>A causes exon skipping	-Cortical blindness (1/2) -Cortical vision impairment (1/2) -DD/ID (2/2) -Epilepsy (2/2) -Secondary microcephaly (2/2)	1298330, 522806	(Ramadesikan et al., 2022)
			M	c.1A>T c.846G>A	p.M1? p.L282	Comp. Het	EOP	progressive cerebral/cerebellar atrophy				
1	1	Chinese	F	c.701 + 1G>A c.194A>G	-p.N65S	Comp. Het	EOP	High signal intensities in bilateral pallidum at 5 months Thin corpus callosum at 16 months Slight enlargement of the ventricles bilaterally at 3 years and deepening of frontotemporal sulci bilaterally	No	-DD/ID -Hypotonia -Left esotropia -Seizure-like symptoms	3609131, 3590478	(Rong et al., 2021)
1	1	Chinese	Unknown	c.628C>T c.770T>C	p.Q210X p.V257A	Comp. Het	LOP	Normal at 28 y; progressive cerebellar atrophy	No	-Ataxic gait -Bradykinesia -Dysphagia -DD/ID -Limbs hypertonia -Slurred speech	3590474, 4884334	(Zhao et al., 2023)
2	2	Iranian	M	c.208T>C	p.C70R	H	EOP	Pontocerebellar atrophy at 3y	No	-Ataxia (1/2) -DD/ID (2/2) -Febrile seizures (2/2) -Language delay (2/2) -Motor delay (1/2) -Neuropathy (1/2) -Nystagmus (1/2) -Strabismus (1/2) -Truncal hypotonia (1/2)	4884335, 638522	(Ghasemi et al., 2024)
			M	c.1274A>G	p.H425R	H	EOP	Pontocerebellar atrophy at 3y				
1	1	Italian	M	c.114 + 3A>G c.440 G>A	-p.S147A	Comp. Het	EOP	w.m.changes at 17 m; progressive pontocerebellar and cerebral atrophy	No	-Convergent strabismus -Epilepsy -Febrile seizure -Non-verbal -Secondary microcephaly -Spasticity -Squint hypertonia following fever -Tetraplegia	984624, 3242529	(Pettinato et al., 2025)
1	1	Iranian	Unknown	c.877G>A	p.A293T	H	EOP	NA	No	-Microcephaly -Motor developmental delay -Spasticity	4884336	(Nejabat et al., 2021)
1	1	Chinese	F	c.194A>G c.896_897insA *Reported as c.896_c.897insA; in (Xuan et al., 2025)	p.N65S p.N299Lfs*2 *Reported as p.N299fs*2 in (Xuan et al., 2025)	Comp. Het	EOP	Thinning of the splenium of the corpus callosum	No	-DD -Epilepsy -Hypotonia -Microcephaly	3590478, 4884328	(Xuan et al., 2025)
2	3	Canadian	M	c.388 + 3A>G	-	H	EOP	Normal	RNA analysis demonstrated aberrant splicing with exon 3 skipping resulting in an out-of-frame transcript	-Ataxia (1/3) -Dysarthria (3/3) -Dystonic features (2/3) -Horizontal nystagmus (1/3) -Microcephaly (1/3) -Motor difficulties (3/3) -Rotatory nystagmus (2/3) -Gait abnormalities (3/3) - Lower-extremity spasticity/spastic diplegia (2/3) - Hyperreflexia without increased tone (1/3)	378569	Current article
			M	c.388 + 3A>G	-	H	EOP	Mild periventricular leukomalacia				
			F	c.388 + 3A>G	-	H	EOP	Normal				
Total: 28	**37**											

DD, developmental delays; ID, intellectual disability; w.m.changes, white matter changes; EOP, LOP.

**FIGURE 1:**
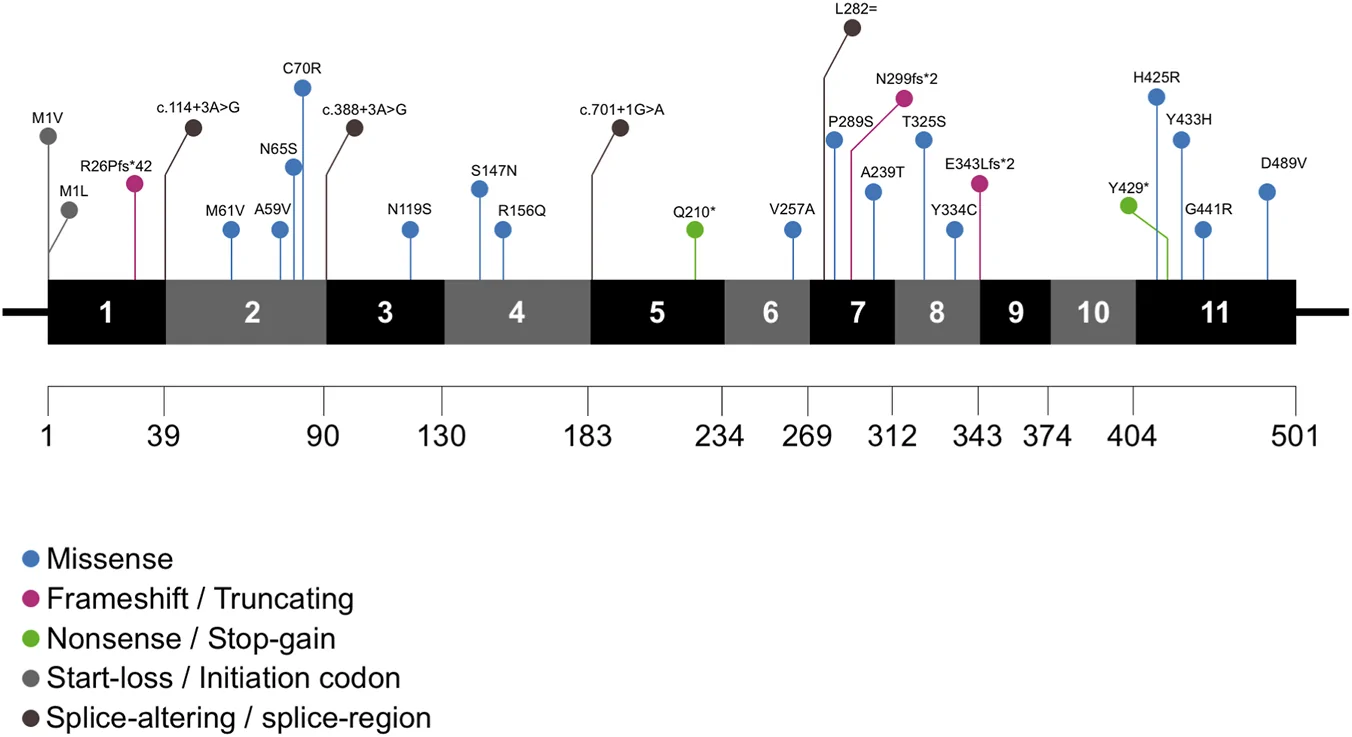
Distribution of reported unique *SEPSECS* variants. Schematic representation of unique *SEPSECS* variant sites reported in individuals with *SEPSECS*-related disease, mapped across the SepSecS protein. Each lollipop represents a unique variant identified in the literature or in the present case series and does not reflect variant frequency or number of affected individuals. Variants are color-coded by predicted molecular consequence, including missense, nonsense, frameshift, start-loss, and splice-altering/splice-region variants. Splice-altering/splice-region variants include intronic splice-site variants and synonymous variants with demonstrated or predicted effects on splicing.

Herein, we report for the first time three affected individuals from two unrelated families originating from the maritime provinces of Canada, all of Acadian descent, who harbor a homozygous intronic *SEPSECS* splice variant. The recurrence of this rare variant in individuals sharing a common ancestral background raises the possibility that this variant may represent a candidate population-specific founder allele. Through detailed clinical, radiological, and genetic characterization, we aim to expand the early-onset phenotypic spectrum of SEPSECS-related pontocerebellar hypoplasia and highlight key features that may facilitate earlier recognition and diagnosis of this rare disorder.

## 2 Materials and methods

### 2.1 Whole exome sequencing

Family 1 (patients 1 and 2): Exome-based testing was performed by CENTOGENE (Rostock, Germany) using the CentoXome® PLUS assay. Genomic DNA was enzymatically fragmented and enriched using DNA capture probes targeting approximately 41 Mb of the human coding exome, including >98% of coding RefSeq regions based on GRCh37/hg19, as well as the mitochondrial genome. Sequencing was performed on an Illumina platform, with at least 20× coverage for >98% of targeted bases. Bioinformatic analysis included alignment to GRCh37/hg19, variant calling, annotation, and comprehensive variant filtering. Rare variants, including those with minor allele frequency <1% in gnomAD and disease-associated variants reported in HGMD®, ClinVar, or CENTOGENE’s internal database, were evaluated in relation to the patients’ phenotype and inheritance pattern. Analysis focused on coding exons and ±10 intronic nucleotides of genes with established gene–phenotype associations, and variants were classified according to ACMG/AMP and ClinGen recommendations.

Family 2 (patient 3): Whole-exome sequencing was performed using the Blueprint Genetics Whole Exome Plus Test (Blueprint Genetics, Helsinki, Finland). The assay targets protein-coding exons, exon–intron boundaries (±20 bp), and selected noncoding/deep intronic variants, and includes sequence analysis, deletion/duplication analysis, and mitochondrial genome analysis. Genomic DNA was processed using hybridization-based target capture followed by paired-end Illumina sequencing. Reads were aligned to GRCh37/hg19 using BWA-MEM, and variant calling was performed using GATK-based algorithms. The test detects single-nucleotide variants, small insertions/deletions up to 220 bp, and single-exon or larger copy-number variants, with annotation using public databases including gnomAD, ClinVar, and HGMD. Analysis focused on established disease-associated genes, with additional evaluation of candidate variants when indicated.

### 2.2 RNA sequencing

Family 1 (patients 1 and 2): Clinical whole exome sequencing with RISE analysis was performed by Fulgent Genetics (El Monte, CA, United States of America). This analysis was performed as part of Fulgent’s clinical WES/RISE package, as RNA analysis was not available as a standalone assay. RNA was extracted from the patient’s blood specimen, and the transcriptome covering the entire coding regions of the genes was analyzed; integrative analysis of DNA and RNA data was performed. FRASER (Find RAre Splicing Events in RNA-seq) tool was used to detect significant aberrant splicing events across the transcriptome, followed by manual BAM inspection. The RNA sequencing analysis of *SEPSECS* variant (NM_016955.4:c.388 + 3A>G; Clinvar variation ID: 378569) showed significant out-of-frame exon skipping when compared to controls (FRASER p-value = 1.13E-06).

Family 2 (patient 3): Research-based RNAseq analysis was performed by a collaborating research team. RNA-seq data was processed and analyzed as previously described (Stark et al., 2025; Hou et al., 2025; Deshwar et al., 2023), followed by manual inspection of BAM files. FRASER was used to detect significant aberrant splicing events. Sashimi plots were generated using ggsashimi for visualization of splicing events (Garrido-Martín et al., 2018).

## 3 Results

### 3.1 Case descriptions–patients 1 and 2

Two male siblings were referred for an initial evaluation at the New Brunswick Medical Genetics Clinic for progressive lower-extremity spasticity consistent with spastic diplegia. The parents are of Acadian descent, without any known consanguinity. An older sibling is reportedly healthy. Maternal family history is significant for spondyloepiphyseal dysplasia, affecting at least two generations, including the patients’ mother with a history of short stature (−3.7 standard deviation), cleft palate and hip tightness.

Patient 1, a 5-year-old male child, with uncomplicated prenatal history, was born at 38 weeks by elective C-section with no significant neonatal complication, no hypoxic events and no abnormal tone. Initial gross motor development was within normal limits with ambulation achieved at 15 months of age. Abnormal spastic gait became noticeable after the age of 2 years with in toeing and toe walking, resulting in difficulty with running, frequent falls and easy fatigability (Figure 2A). Fine motor development was age appropriate and speech was mildly delayed. Behavioral challenges, including frustration related to impaired oral communication were noted. There was no history of seizures or regression. At the age of 4, the patient developed rotatory nystagmus; corrective lenses were prescribed.

**FIGURE 2:**
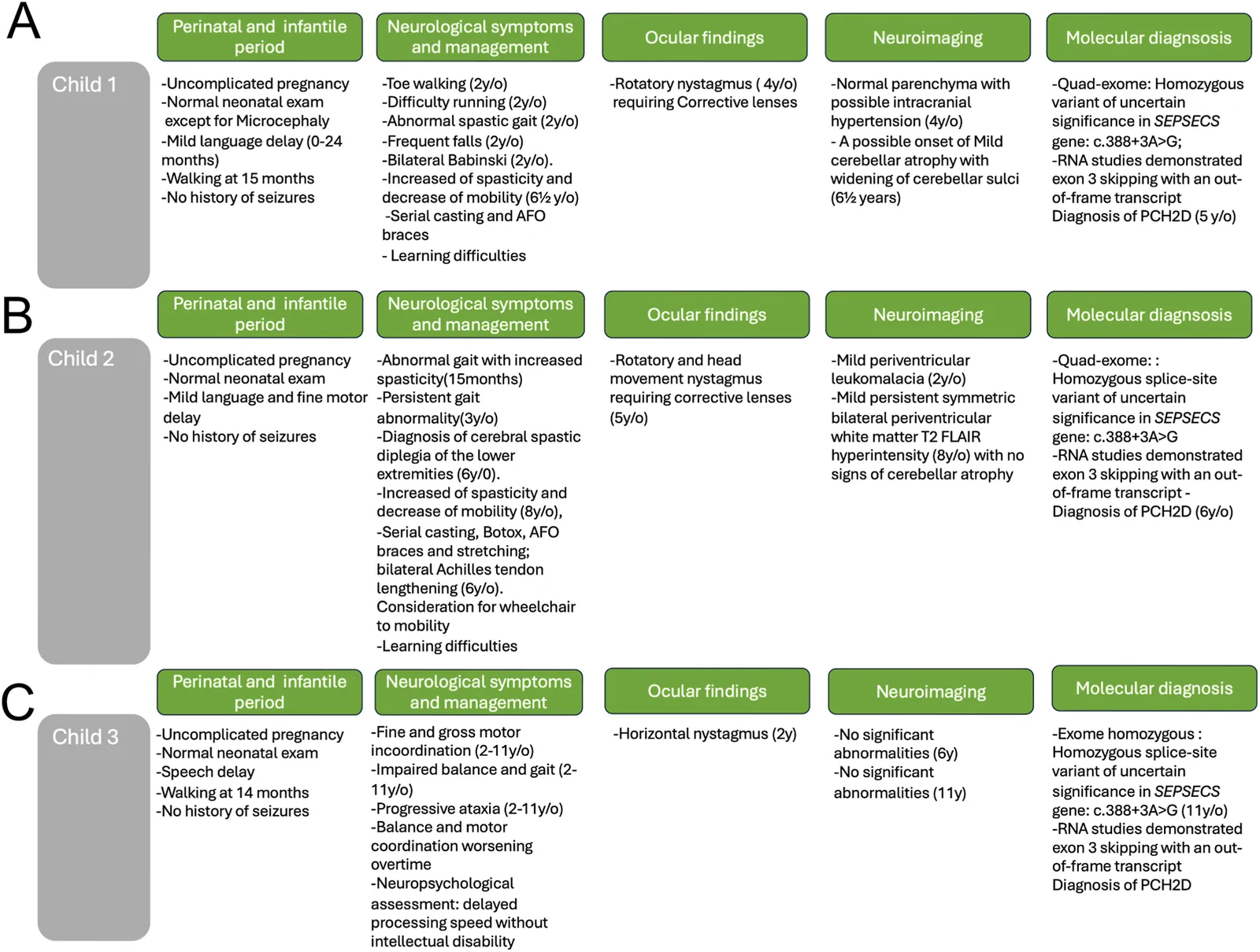
Domain-based clinical summary of the affected individuals. Comparative summary of the main clinical features, investigations, and current status of the three affected individuals with homozygous *SEPSECS* NM_016955.4:388 + 3A>G. Each row represents one affected individual, and findings are organized by clinical domain, including development, motor signs, ocular findings, neuroimaging, and current status/genetics. **(A)**, Patient 1. **(B)**, Patient 2. **(C)**, Patient 3. Ages at onset, assessment, or investigation are indicated in parentheses when available. AFO, ankle-foot orthoses; FLAIR, fluid-attenuated inversion recovery; MRI, magnetic resonance imaging; PCH2D, pontocerebellar hypoplasia type 2D.

On examination, weight was 16.1 kg (10–25th percentile), height 107.6 cm (25–50th percentile), and head circumference of 48.5 cm (∼2nd percentile). Facial features included mild bilateral epicanthic folds and mildly thickened earlobes. Musculoskeletal exam revealed joint hyperlaxity of fingers and knees, no scoliosis or pectus deformity. Neurologically, there was increased tone in the lower extremities, more pronounced on the left, brisk and symmetric deep tendon reflexes, bilateral Babinski signs, and a spastic gait pattern with compensatory circumduction, mild scissoring, and bilateral toe walking.

Lower-extremity and pelvic X-rays performed were normal. At 4 years old, brain MRI showed normal parenchyma, with possible intracranial hypertension but no Chiari malformation. Follow-up brain MRI performed in 2026 to assess for progressive cerebral or cerebellar atrophy demonstrated at least mild cerebellar atrophy, with widening of the cerebellar sulci most pronounced in the vermis, compared with the prior examination. Supratentorial brain volume remained within normal limits. Evaluations by neurologist and physical medicine specialist confirmed the diagnosis of spastic diplegia of the lower extremities with dystonic features. The patient required focal chemodenervation, serial casting and ankle-foot-orthoses.

Patient 2 is the older sibling of patient 1, a male child of 6.5 years of age. Prenatal history was uncomplicated. He was born at 38 weeks gestation by elective Caesarean section. Initially, the patient had a normal motor development. Abnormal gait and increased lower-extremity tone were noted after 15 months (Figure 2B). They also had a history of mild fine motor delay and received speech therapy for articulation. There is no history of seizures or regression. The patient developed Rotatory nystagmus at the age of 5. Previous ophthalmologic evaluation revealed head-movement nystagmus.

On examination, weight was 22.5 kg (50–75th percentile), height 115 cm (10–25th percentile), head circumference 51 cm (∼50th percentile). Facial features were similar to his brother: mild bilateral epicanthic folds, mildly tented upper lip, mildly prominent ears, and high narrow palate. Neurologically, increased lower-extremity tone (left > right), brisk reflexes with 1–2 beats of clonus bilaterally, and a spastic gait pattern with compensatory circumduction and mild scissoring.

Investigation including renal and liver function, calcium phosphate, iron studies and C-reactive protein, very long chain fatty acids and creatinine kinase levels were all normal. Lower-extremity and pelvic X-rays, as well as spine MRI, were performed and did not reveal any significant findings. Brain MRI at 2 years old, revealed mild periventricular leukomalacia. Follow-up brain MRI performed in 2026 did not demonstrate progressive cerebral or cerebellar atrophy. Mild bilateral T2/FLAIR white matter hyperintensities, most pronounced around the occipital horns, were stable compared with prior imaging and showed no cystic change. Evaluation by a physical medicine specialist and neurologist concluded that he had cerebral spastic diplegia of the lower extremities in the absence of perinatal insult. Patient required serial casting, focal chemodenervation, ankle–foot orthoses, bilateral Achilles tendon lengthening, and daily stretching.

### 3.2 Case description–patient 3

Patient 3 is an 11-year-old female of Acadian ancestry referred to Medical Genetics in Halifax, Nova Scotia for progressive ataxia, gait abnormalities, nystagmus, hypotonia and school difficulties. She was born following an uncomplicated pregnancy at 41 weeks gestation by induced vaginal delivery with no significant neonatal complications. Feeding difficulties in infancy were attributed to tongue-tie. Early development was largely normal with independent walking at 14 months, although speech dysfluency was noted early and worsened over time (Figure 2C).

Fine and gross motor difficulties became apparent between 2 and 3 years of age, with progressive impairment in coordination and balance. She had difficulties with running, stairs and fine motor tasks such as brushing her hair and school-related activities. Progression became particularly evident when she lost the ability to run uphill. Parents also noted non progressive horizontal nystagmus around the age of 2 years and ophthalmologic evaluation led to prism glasses. She was assessed by neurology and physiatry after concerns for possible mild cerebral palsy. Findings demonstrated tightness in the hips, ankles, hamstrings, inwards turning of the ankles and pes planus. Her balance and coordination worsened over time. Neuropsychological evaluation demonstrated delayed processing speed without intellectual disability. Anxiety and separation anxiety were also noted.

Previous investigations including urine organic acids, plasma and urine amino acids, ammonia, lactate, venous blood gas and routine biochemical studies were unremarkable aside from mildly low glucose. Brain MRI performed in 2019 for nystagmus and repeated in 2024 for progression of neurological symptoms showed no significant abnormalities. Cardiac evaluation including electrocardiogram and echocardiogram was normal.

Family history revealed that both parents, although not known to be related, originate from the same small Acadian community in New Brunswick. On examination, height was 139,4 cm (3rd-10th percentile), weight 27.1 kg (3rd percentile) and head circumference was within the normal range. No dysmorphic features were identified. Speech was slow with mild dysarthric features. Gait was stiff with mild hip flexion and impaired knee lift during running. Balance was impaired, requiring support while standing on one foot and occasionally touching walls while walking. Muscle strength was preserved. Neurological examination demonstrated bilateral lower extremity hyperreflexia without clonus or increased tone, horizontal nystagmus and dysdiadochokinesis while finger-to-nose, heel-to-shin and Romberg testing were largely preserved. Overall findings raised concern for a progressive neurogenetic disorder.

### 3.3 Investigations–patients 1 and 2

At the initial evaluation in the NB Medical Genetics Clinic (2025), patients 1 and 2 were diagnosed with spastic diplegia of the lower extremities, with possible dystonic features in patient 2. Both siblings exhibited mild expressive language and fine motor delays. A genetic etiology was strongly suspected, with consideration given to a hereditary form of spastic paraplegia with or without dystonia; a skeletal dysplasia or inborn error of metabolism was considered less likely. Autosomal dominant and X-linked modes of inheritance were initially considered. Accordingly, metabolic investigations, including urine organic acids and plasma acylcarnitine profiles, were completed and were within normal limits.

CentoXome® PLUS assay (CENTOGENE; Rostock, Germany) was requested in both siblings, with parental testing included to improve diagnostic yield. The results revealed a homozygous variant of uncertain significance (VUS) in the *SEPSECS* gene**, c.388+3A>G** (Clinvar variation ID: 378569), in both siblings. Parents were found to be heterozygous for this variant.

RNA analysis was performed by Fulgent Genetics to evaluate the pathogenicity of the *SEPSECS* c.388 + 3A>G variant and to confirm the diagnosis of PCH2D. Results demonstrated aberrant splicing with exon 3 skipping, generating an out-of-frame transcript consistent with a loss-of-function mechanism (Figure 3A). RNA analysis revealed exon 3 skipping in approximately 77% of informative reads, with 20 reads supporting exon 3 skipping and 6 reads supporting normally spliced transcript. This aberrant splicing event was statistically significant by FRASER analysis (p = 1.13 × 10^−6^). In the heterozygous carrier parents, low-level exon skipping was observed, with 3/18 reads (∼16.6%) in the mother and 3/17 reads (∼17.6%) in the father, whereas no exon 3 skipping was observed in 32 control samples (Figure 3A).

**FIGURE 3:**
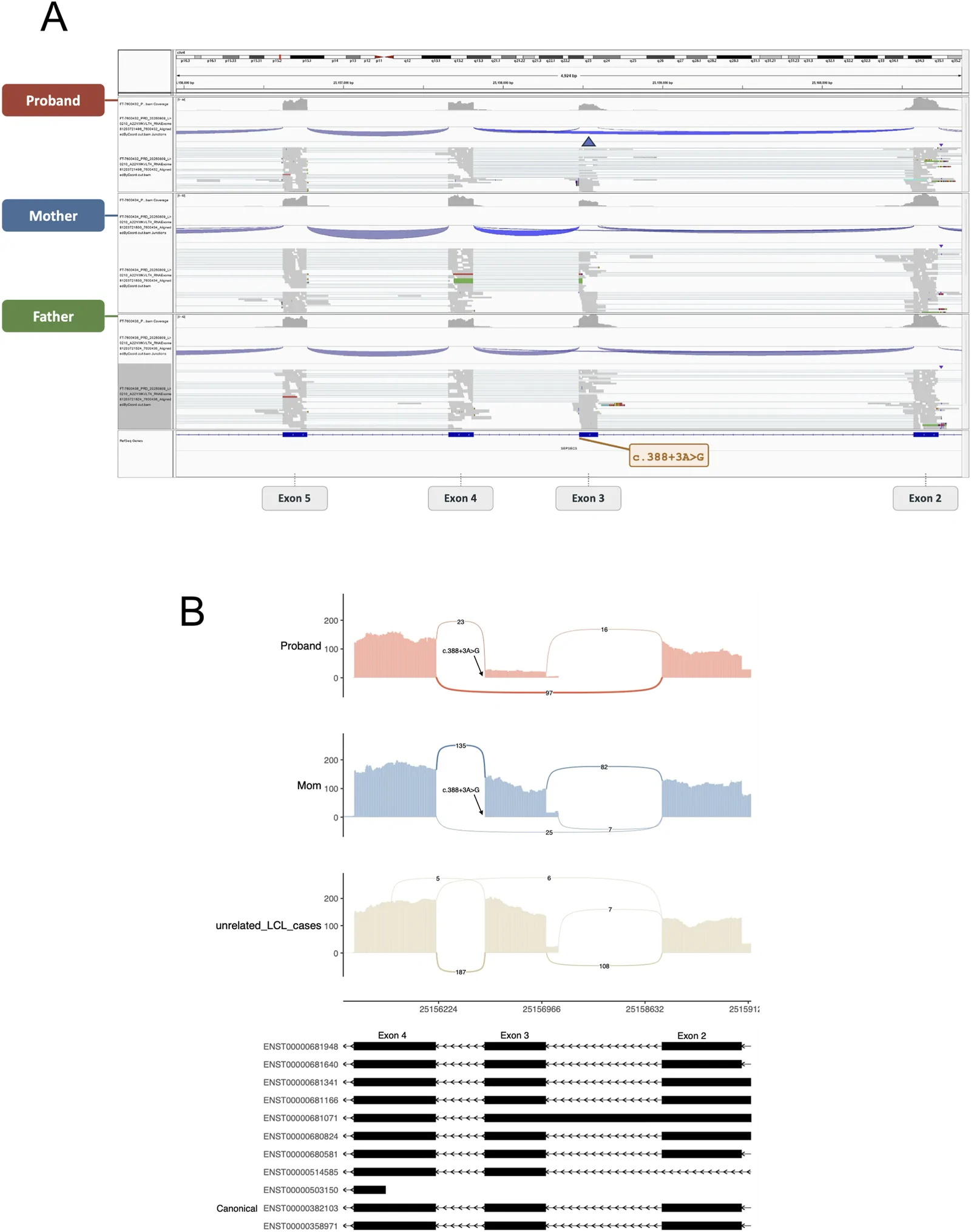
RNA-seq visualization of the *SEPSECS* locus: **(A)** Family 1: RNA-seq visualization of the *SEPSECS* locus at chr4:25,155,600–25,160,900 (GRCh37) in an affected individual from Family 1 carrying the homozygous NM_016955.4:388 + 3A>G variant. Coverage, splice junctions, aligned reads, and the RefSeq gene track are shown. Grey bars represent RNA-seq read coverage, blue arcs represent junction-spanning reads, and the RefSeq gene track indicates *SEPSECS* exon structure. The c.388 + 3A>G variant affects the exon 3 splice donor region, with exon 3 skipping visualized by a long-range junction bridging exon 2 to exon 4. **(B)** Family 2: Sashimi plot of *SEPSECS* alternative splicing at exon 3. The variant c.388 + 3A>G is indicated by the arrow. RNA-seq coverage tracks are displayed for the proband (top), maternal heterozygous carrier (middle), and a cohort of unrelated controls (bottom; n = 40, representing the median read depth across individuals). Only splice junctions supported by a minimum of 5 reads are visualized.

The variant was reclassified as Pathogenic using the following ACMG/AMP criteria: PVS1_Strong, PM3_Strong, PM2, and PP1. Functional RNA evidence was incorporated as PVS1_Strong, as RNA-seq demonstrated predominant out-of-frame exon skipping consistent with a loss-of-function effect. PM3_Strong was applied based on a total of four independent reported affected individuals/families. This includes previously reported compound heterozygous individuals carrying c.388 + 3A>G in trans with another pathogenic SEPSECS variant (PMID:25590979; PMID:33528536), each contributing 0.5 points under the recessive PM3 framework, as well as two unrelated affected families in the present study with homozygous c.388 + 3A>G, collectively contributing additional evidence. Together, these observations support PM3_Strong. PM2 was applied based on rarity in population databases, with an allele frequency of 0.00006941 (103/1,483,956) in gnomAD v4.1. Finally, PP1 was applied based on segregation evidence from the affected sibling pair reported in this study. Consequently, a molecular diagnosis of pontocerebellar hypoplasia type 2D was established in both patients.

### 3.4 Investigation–patient 3

Given concern for a hereditary progressive neurological disorder, exome sequencing was pursued and identified a homozygous likely pathogenic splice variant in *SEPSECS*, c.388 + 3A>G (Clinvar variation ID: 378569), the same variant identified in patients 1 and 2. Both parents were heterozygous for this variant.

Research RNA studies were subsequently performed to further characterize the variant and demonstrated exon 3 skipping resulting in an out-of-frame transcript consistent with aberrant splicing and a loss-of-function mechanism. Exon 3 skipping was observed in 10% of maternal RNA-seq reads (25/249 reads; FRASER p-value = 0.0096967), consistent with heterozygosity, compared with 71% of RNA-seq reads (97/136 reads; FRASER p-value = 1.7798 × 10^−6^) in patient 3, indicating a substantially higher proportion of exon 3 skipping in the affected individual (Figure 3B). These findings support the pathogenicity of the variant and confirm a molecular diagnosis of *SEPSECS*-related disease.

Although the initial laboratory-reported classifications differed across patients, with c.388 + 3A>G reported as a VUS in Patients 1 and 2 and as likely pathogenic in Patient 3, integration of RNA evidence demonstrated a concordant splice-altering effect in all three affected individuals. The variant was therefore interpreted at the variant level as pathogenic according to ACMG/AMP and ClinGen SVI splicing recommendations.

## 4 Discussion

Pontocerebellar hypoplasia type 2D (PCH2D) remains an ultra-rare neurodevelopmental disorder, with approximately 34 reported individuals from 26 families worldwide (Table 1). Our report expands this limited body of literature by describing three affected individuals from two Acadian families harboring the same intronic *SEPSECS* variant, NM_016955.4:c.388 + 3A>G, and by providing functional RNA evidence supporting its pathogenic classification.

The affected patients in our series presented with early-onset lower-extremity spasticity, progressive gait abnormalities, and mild neurodevelopmental delay. *SEPSECS*-related PCH2D appears to encompass at least three broad clinical patterns: severe early neonatal presentation, often characterized by profound microcephaly, refractory epilepsy, progressive cerebellar and cerebral atrophy, respiratory failure, and early mortality in some cases; a milder attenuated infantile form; and a childhood LOP, with the latter two categories showing more slowly progressive neurological involvement (Zhao et al., 2023; van Dijk et al., 2018; Iwama et al., 2016; Nicita et al., 2022; Ramadesikan et al., 2022). In this context, we use the term “attenuated infantile-onset phenotype” descriptively to refer to individuals whose symptoms begin in infancy or early childhood, whose clinical course is less severe than classical severe neonatal presentation. The present patients fit this category because symptoms began in infancy or early childhood, yet none had seizures or rapid neurodevelopmental regression, cognitive/adaptive function was comparatively preserved, and early neuroimaging findings were absent or subtle. However, follow-up MRI in Patient 1 later demonstrated at least mild cerebellar atrophy, with widening of the cerebellar sulci most pronounced in the vermis, whereas follow-up imaging in Patient 2 remained stable without evidence of progressive cerebral or cerebellar atrophy. Patient 3 also underwent repeat brain MRI in 2024, which did not demonstrate pontocerebellar hypoplasia. Thus, the term “attenuated infantile-onset phenotype” is intended to describe onset of symptoms in infancy with reduced overall severity compared to classic severe neonatal presentation and does not imply uniform neuroimaging findings or identical progression across affected individuals.

Together, these findings suggest that neuroimaging abnormalities in this cohort were absent or subtle initially and may evolve over time, while remaining less severe than those reported in classical early-onset PCH2D. This also raises a nosological consideration: although PCH2D is classically defined by pontocerebellar hypoplasia, molecularly confirmed *SEPSECS*-related disease may not show overt pontocerebellar hypoplasia on initial MRI. Therefore, absence of classical pontocerebellar hypoplasia, particularly early in the disease course, should not exclude consideration of *SEPSECS*-related disease when the clinical and molecular findings are supportive. These observations suggest that *SEPSECS*-related PCH2D may encompass broader neuroimaging variability than implied by the diagnostic label alone.

This observation aligns with increasing recognition of phenotypic heterogeneity in *SEPSECS*-related disease, for which consistent genotype–phenotype correlations have not been established. Pathogenic variants of varying types are distributed throughout the gene, and individuals harboring similar variant classes may demonstrate variability in severity and/or progression over time (Iwama et al., 2016; Nicita et al., 2022). In our cohort, the comparatively attenuated infantile-onset phenotype may be related to the specific molecular consequence of the homozygous c.388 + 3A>G variant. RNA analyses demonstrated aberrant splicing with exon 3 skipping, generating an out-of-frame transcript consistent with a loss-of-function mechanism. However, the splice defect appeared incomplete, with evidence of residual normally spliced transcript. The detection of residual normally spliced transcript is consistent with a partial or “leaky” splice defect at the RNA level. This finding provides a plausible explanation for why some residual SepSecS function could be preserved; however, residual SepSecS protein levels, enzymatic activity, and downstream selenoprotein biosynthesis were not directly assessed. Therefore, any contribution of residual normally spliced transcript to the milder clinical presentation remains hypothetical. The zygosity of the c.388 + 3A>G variant may also be relevant, although this interpretation remains speculative. Two previously reported individuals carrying c.388 + 3A>G in compound heterozygosity with another pathogenic *SEPSECS* variant had a more classical PCH2D presentation (Zhu et al., 2015; Moreno-De-Luca et al., 2021). In contrast, all affected individuals in the present series are homozygous for c.388 + 3A>G and appear to have a comparatively milder infantile-onset phenotype as opposed to the severe neonatal form. One possible explanation is that c.388 + 3A>G behaves as a hypomorphic splice allele, with residual normally spliced transcript potentially allowing partial preservation of SepSecS function. However, this remains a hypothesis, as residual protein function and downstream selenoprotein biosynthesis were not directly measured. Moreover, clinical variability was observed within the present case series, including between the two affected siblings from the same family. Although both siblings shared the same homozygous *SEPSECS* c.388 + 3A>G variant and overlapping features of early-onset lower-extremity spasticity, gait abnormalities, and nystagmus, Patient 2 appeared more affected clinically, with earlier motor concerns and mild periventricular leukomalacia on brain imaging. This intrafamilial variability further suggests that the milder phenotype cannot be explained by residual splicing alone and that additional genetic, epigenetic, environmental, developmental, or tissue-specific modifiers may influence disease expression.

A limitation of this study is that clinical phenotyping was retrospective and based on available clinical documentation rather than a standardized prospective neurological assessment. As a result, some domains, including detailed strength testing, peripheral involvement, bulbar and respiratory function, and standardized adaptive functioning, could not be systematically compared across all three individuals.

More broadly, this case series highlights the importance of transcript-level analyses for interpreting intronic variants (Oh et al., 2024). The majority of previously reported pathogenic *SEPSECS* variants are missense or truncating changes affecting conserved regions of the enzyme, whereas intronic or splice-altering variants remain comparatively underrepresented and are not always functionally confirmed at the RNA level.

Among reported splice-altering *SEPSECS* variants, disease severity appears variable, across the limited number of individuals described to date (Ramadesikan et al., 2022; Zhu et al., 2015; Arrudi-Moreno et al., 2021; Rong et al., 2021; Pettinato et al., 2025; Moreno-De-Luca et al., 2021). However, these observations should be interpreted cautiously, as several variants were reported in compound heterozygosity with different second alleles, making it difficult to determine the independent contribution of each splice-altering variant to disease severity. For example, the synonymous splice-altering variant c.846G>A, p. Leu282 = , was reported in association with a severe phenotype but occurred in compound heterozygosity with a start-loss variant. Similarly, other splice-site variants, including c.701 + 1G>A and c.114 + 3A>G, have been reported in distinct allelic and clinical contexts (Ramadesikan et al., 2022; Pettinato et al., 2025). Therefore, while transcript-level consequences and the degree of residual normal splicing may contribute to phenotypic variability, available data remain insufficient to establish clear genotype-phenotype correlations based on variant class, splice-site position, or zygosity alone. Because RNA analysis in our study was performed in blood-derived samples, the degree of exon 3 skipping in disease-relevant neural tissues cannot be directly assessed and tissue-specific differences in splicing, transcript stability, or RNA surveillance cannot be excluded. In addition, residual SepSecS protein and downstream selenoprotein biosynthesis were not directly assessed, as those functional assays were outside the scope of the present report. Nevertheless, the detection of residual normally spliced transcript supports the possibility that c.388 + 3A>G does not completely abolish normal *SEPSECS* splicing in blood-derived RNA. Whether this residual transcript results in measurable residual SepSecS protein function or contributes to clinical attenuation remains to be determined.

An additional point highlighted by this case series is the variability in variant interpretation between diagnostic laboratories. The c.388 + 3A>G variant was initially classified as a VUS in Patients 1 and 2, whereas it was classified as likely pathogenic in patient 3. These differences likely reflected laboratory-specific classification frameworks, available evidence at the time of reporting and access to internal or published case data. Following RNA studies demonstrating concordant splice-altering effect involving exon 3, the final variant-level interpretation was harmonized as pathogenic across all three affected individuals. This reinforces the importance of periodic variant reinterpretation and integration of orthogonal evidence, particularly RNA studies, when evaluating non-canonical splice-site variants.

Finally, the recurrence of the same rare intronic *SEPSECS* variant in three affected individuals from two unrelated families of Acadian raises the possibility that c.388 + 3A>G may represent a candidate Acadian founder allele. The Acadian population represents a genetically distinct founder population having experienced a severe genetic bottleneck during the Grand Dérangement of 1755 (Robichaud et al., 2022). This demographic history which is composed of a small number of original founders creates precisely the conditions that favours the enrichment of rare pathogenic alleles through genetic drift leading to disproportionate burden of autosomal recessive diseases (Marafi, 2024). Indeed, Acadians exhibit reduced genetic diversity and loss of rare haplotypes compared to their European source population (Moreau et al., 2009). The first carrier screening study in Acadians of South-East New Brunswick identified carrier status for 28 autosomal recessive disease (Robichaud et al., 2022) and several Acadian-specific founder mutations have been formally demonstrated. Given this well-established pattern of founder mutations in the Acadian population, the hypothesis that c.388 + 3A>G represents an additional Acadian founder allele is both plausible and consistent with the available evidence. This founder-effect hypothesis remains preliminary. In fact, analysis of regions of homozygosity surrounding *SEPSECS*, assessment of shared haplotype length, variant-age estimation, and screening of additional individuals of Acadian ancestry would be required to determine whether c.388 + 3A>G is inherited on a shared ancestral haplotype to support the hypothesis of an Acadian founder allele. If confirmed, this would have important implications for targeted carrier testing, early diagnosis, and genetic counselling in individuals of Acadian ancestry.

## 5 Conclusion

This case series expands the molecular and clinical spectrum of *SEPSECS*-related PCH2D by (1) providing functional RNA confirmation of a non-canonical intronic splice variant, (2) documenting phenotypic variability in early-onset presentations, and (3) raising the possibility of a candidate founder allele in individuals of Acadian descent. These findings contribute to improved recognition of *SEPSECS*-related disease and highlight the importance of transcript-level analyses in rare neurogenetic disorders.

## Data Availability

The original contributions presented in the study are publicly available. This data can be found in the Clinvar repository under the accession numbers SCV007664812 to SCV007664822.
